# Structure and integration of specialty palliative care in three NCI-designated cancer centers: a mixed methods case study

**DOI:** 10.1186/s12904-023-01182-9

**Published:** 2023-05-16

**Authors:** Karen E. Schifferdecker, Rebecca L. Butcher, Genevra F. Murray, Kristin E. Knutzen, Nirav S. Kapadia, Gabriel A. Brooks, Garrett T. Wasp, Susan Eggly, Laura C. Hanson, Gabrielle B. Rocque, Amanda N. Perry, Amber E. Barnato

**Affiliations:** 1 The Dartmouth Institute for Health Policy and Clinical Practice at Geisel School of Medicine, Dartmouth College, WTRB Level 5, 1 Medical Center Drive, Lebanon, NH 03756 USA; 2 New York University School of Global Public Health, 708 Broadway, New York, NY 10003 USA; 3 Emory Rollins School of Public Health, 1518 Clitton Rd. NE, Atlanta, GA 30322 USA; 4 Dartmouth Health Department of Medicine, One Medical Center Drive, Lebanon, NH 03756 USA; 5 Wayne State University School of Medicine, Karmanos Cancer Institute, Mid-Med Lofts, Suite 3000, 87 E Canfield, Detroit, MI 48201 USA; 6 University of North Carolina-Chapel Hill School of Medicine, 5003 Old Clinic, CB# 7550, Chapel Hill, NC 27599 USA; 7 University of Alabama at Birmingham, 500 Second Street South, Birmingham, AL 35233 USA

**Keywords:** Specialty palliative, Care integration

## Abstract

**Introduction:**

Early access to specialty palliative care is associated with better quality of life, less intensive end-of-life treatment and improved outcomes for patients with advanced cancer. However, significant variation exists in implementation and integration of palliative care. This study compares the organizational, sociocultural, and clinical factors that support or hinder palliative care integration across three U.S. cancer centers using an in-depth mixed methods case study design and proposes a middle range theory to further characterize specialty palliative care integration.

**Methods:**

Mixed methods data collection included document review, semi-structured interviews, direct clinical observation, and context data related to site characteristics and patient demographics. A mixed inductive and deductive approach and triangulation was used to analyze and compare sites’ palliative care delivery models, organizational structures, social norms, and clinician beliefs and practices.

**Results:**

Sites included an urban center in the Midwest and two in the Southeast. Data included 62 clinician and 27 leader interviews, observations of 410 inpatient and outpatient encounters and seven non-encounter-based meetings, and multiple documents. Two sites had high levels of “favorable” organizational influences for specialty palliative care integration, including screening, policies, and other structures facilitating integration of specialty palliative care into advanced cancer care. The third site lacked formal organizational policies and structures for specialty palliative care, had a small specialty palliative care team, espoused an organizational identity linked to treatment innovation, and demonstrated strong social norms for oncologist primacy in decision making. This combination led to low levels of specialty palliative care integration and greater reliance on individual clinicians to initiate palliative care.

**Conclusion:**

Integration of specialty palliative care services in advanced cancer care was associated with a complex interaction of organization-level factors, social norms, and individual clinician orientation. The resulting middle range theory suggests that formal structures and policies for specialty palliative care combined with supportive social norms are associated with greater palliative care integration in advanced cancer care, and less influence of individual clinician preferences or tendencies to continue treatment. These results suggest multi-faceted efforts at different levels, including social norms, may be needed to improve specialty palliative care integration for advanced cancer patients.

**Supplementary Information:**

The online version contains supplementary material available at 10.1186/s12904-023-01182-9.

## Background

Early access to specialty palliative care improves outcomes for patients with advanced cancer, including frequency of advanced care planning, higher quality of life, lower symptom burden, greater patient and caregiver satisfaction, lower end-of-life (EOL) health care utilization [1] and, in some cases, overall survival [2]. As a result, the American Society of Clinical Oncology guidelines state that “inpatients and outpatients with advanced cancer should receive dedicated palliative care services, early in the disease course, concurrent with active treatment” and that “referral of patients to interdisciplinary palliative care teams is optimal”[3].

Interest in specialty palliative care teams for patients with cancer has led to numerous models of palliative care delivery [4], summaries of palliative care principles and best practices [3] and indicators of palliative care integration [5]. Despite the proliferation of models and guides for specialty palliative care integration, significant variation in palliative care delivery exists due to capacity and resources for delivering palliative care, including who is involved, when it is introduced and how it is integrated into other aspects of cancer treatment [6]. Given the variation in palliative care integration and difficulty in creating rigorous experimental designs, Aoun and Nekolaichuk [7] suggest that improving the evidence for palliative care integration should include studies designed to take “into account the unique qualities and culture of each setting” including mixed methods and qualitative approaches.

This study aims to better define and compare integration of specialty palliative care by comparing palliative care service delivery for advanced cancer patients at three National Cancer Institute (NCI) designated cancer centers. Using existing site-level context data and in-depth qualitative data, we examine and compare the organizational structures and processes related to engagement of specialty palliative care, and the organizational, clinician, and team-based practices and norms that may support or hinder its integration across the three sites. In the end, we propose a middle range theory that suggests a causal model of factors and interactions associated with the integration of specialty palliative care that could be further tested.

## Methods

We collected data from July 2019 through March 2020 as part of a larger study to explore the mechanisms underlying variation in EOL treatment intensity for minority patients [8–11]. In the larger study, we purposely selected minority-serving centers and examined National Quality Forum (NQF) endorsed EOL quality metrics [12] at these centers among Medicare fee-for-service patients with advanced cancer. The NQF EOL quality metrics we examined included chemotherapy in the last 2 weeks of life (NQF #0210), intensive care unit (ICU) admission in the last 30 days of life (NQF #0213), hospice admission in the last 30 days of life (NQF #0215), and hospice length of stay less than 3 days (NQF #0216). In selecting sites, we considered these multivariable metrics individually, in pairs, and using multivariate data visualizations [13].

The Dartmouth College Committee for the Protection of Human Subjects at Dartmouth College (#00031129) and the Wayne State University Institutional Review Board (#055219B3E) reviewed and approved the study, which was designated minimal risk. The other two participating sites delegated to the Dartmouth College Committee for the Protection of Human Subjects through a reliance agreement. Our study was carried out in accordance with the Declaration of Helsinki. A certificate of confidentiality was also obtained for this study from the National Institutes of Health.

Details of the overall study and methods, which we used for this more focused study of palliative care integration, are described in detail by Knutzen et al [9]. Below we briefly summarize and describe the current study.

### Site selection

We used 2016 Medicare claims data analyses to identify cancer centers that were either NCI-designated cancer centers and/or members of the National Comprehensive Cancer Network (NCCN) and that served at least 15% Black patients with advanced cancer to meet our definition of minority-serving [8]. In the overall case study, we aimed to recruit and compare a heterogeneous sample of six sites to maximize variation in geographic region and EOL treatment intensity. However, due to the COVID-19 pandemic, we only visited three of the six selected sites which included an urban center in the northern Midwest (Site A) and two centers in the southeast (Sites B and C). We categorized all three centers as having better than average EOL quality based on the Medicare claims analyses of NQF EOL metrics.

### Case study design

We used a mixed-methods, case study design which included semi-structured interviews with leaders, clinicians, patients and caregivers; observations of clinical encounters, tumor boards and other relevant care conferences during multi-day site visits; and review of site context data, including patient volume, demographics, and policies related to advanced cancer care and end of life planning. Per our Institutional Review Board protocol, clinicians and leaders were sent an information sheet and informed consent was obtained via email prior to interviews. Patients and caregivers were given information about the study during their observation and provided verbal informed consent at the start of their interviews. Providers and staff who were observed were introduced to the research team members before observation, informational flyers hung around the clinics, and providers and staff could opt out of being included in observation notes.

Details of the topics and focus for each data collection method, previously described in-depth [9], included an exploration of the following: organizational resources, programs, and policies related to EOL and specialty palliative care; structures and capacity for palliative and hospice care; decision-making related to treatment, including timing of decisions to refer to specialty palliative care and/or discontinue disease-directed therapies, and social norms and clinician orientation to advanced cancer care (see Additional File 1-leader interview guide). The data sources used in this analysis included all but patient and caregiver interviews.

In clinician interviews we used a mix of general questions and vignette-based exploration of considerations when treating advanced cancer patients (see Additional Files 2 and 3-clinician interview guide; patient vignettes). The vignettes were brief patient cases developed by our partner oncologists (NSK, GAB, GTW) where disease-directed treatment benefits were waning and symptom burden was increasing. During interviews, we asked clinicians to read vignettes and answer questions to explore if and how the clinician discussed palliative care as a course of action compared to the continuation of disease-directed treatment, including their rationale and decision points. Interviews and observations were audio recorded and documented with extensive notes respectively, transcribed, and de-identified using identification codes for sites and participant roles to facilitate linkage and comparison of data across sources.

*Data analysis*: All data were uploaded for coding and analysis in Dedoose, a qualitative and mixed methods data analysis platform [14]. We analyzed qualitative data using a mixed inductive and deductive approach [15, 16] and triangulation, a process to crosscheck data and assess saturation of findings. We used two of the four major types of triangulation described by Denzin [17]: methodological triangulation and investigator triangulation. We used methodological triangulation by comparing results across different data sources and methods (e.g., interviews, observations), and investigator triangulation by always having at least three researchers involved in both data collection and analyses (GFM, KEK, AEB) and involving other research team members in review and discussion of findings across our methods. The main outcome of interest in our analysis was specialty palliative care integration in advanced cancer care, which we define as factors that appeared to either support or hinder its involvement and delivery.

**Fig. 1:**
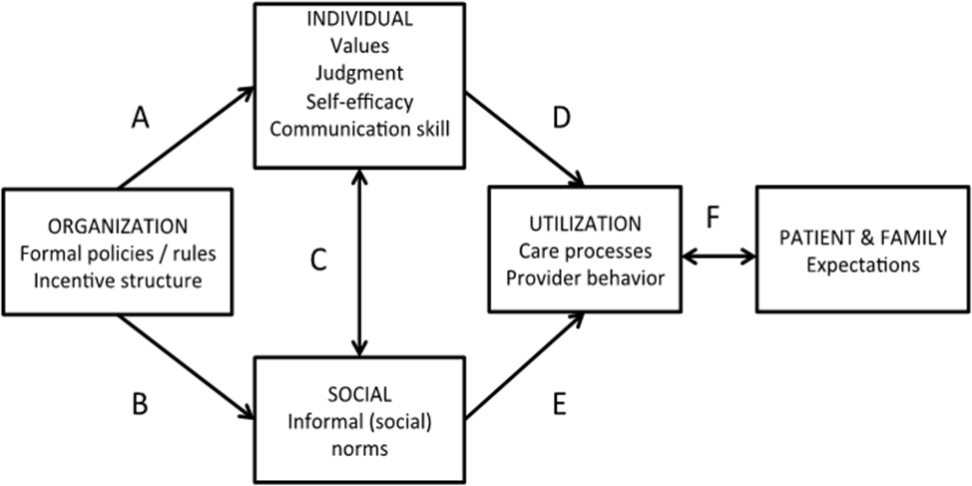
Conceptual model for palliative care utilization

For our mixed inductive and deductive codebook development, we first defined descriptor codes inductively which are high-level categories related to each data set (e.g., interview, observation). Examples include the site ID, specialty, role (e.g., leader) of person being interviewed, and length of time with patient. These descriptor codes allow data to be filtered and compared across the different descriptor types. Next, three research team members (KES, RLB, GFM) used a conceptual framework developed by our study’s principal investigator (AEB; see Fig. 1) based on her prior research related to norms in palliative care [18, 19] to pre-define additional deductive codes. The framework integrates concepts from individual and organizational behavioral theories [20, 21] and focused on three primary levels of potential influence—organizational structures, social norms, and individual characteristics and skills.

We defined organization-level structures as the personnel, systems, physical space, and rules in place that dictate or influence how activities are done related to palliative care and EOL. These include policies, staffing roles and hierarchies, responsibilities and actions conducted through regular meetings or written processes and practices. Social norms are defined as rules about which there is at least some degree of consensus, enforced through social sanctions or support [22]. Social norms may be formal (e.g., written policies or guidelines), or informal, more unspoken and implicit between members of groups. In our analysis, we included formal norms as part of the organization-level structure and defined social norms as being informal, implicit group-based social norms. At the level of the individual, we considered the attitudes, beliefs and experiences of participants shaping behaviors and actions.

Starting with the initial codebook, a core coding group (three coders) expanded and refined the codebook through iterative, inductive coding with focused review by all other team members, and reliability checks by the lead coder (RLB). Questions or disagreements in both the coding process and code definitions were discussed and often resolved within the core coding group. However, some questions were brought to the full research team which included clinicians working in oncology and palliative care for discussion and resolution. Throughout the coding process, code application analyses and code memos were used by two team members (KES, RLB) to begin to identify patterns and key ideas.

Our final codebook included 268 codes, of which 227 were specifically applicable to this more focused study of palliative care integration and structures. Codes included sets for clinical decisions (e.g., treatment selection, treatment cessation); clinical actions (e.g., interventions that happened or not); decision-making influences; interpersonal communications and dynamics (e.g., staff-clinician, clinician-clinician); organization-level factors; social norms; clinician-level attitudes, beliefs, and satisfaction; observation-related codes (e.g., provider orientation to patient, presence/absence of goals of care discussions); and patient attribute codes (e.g., social support, socioeconomic factors).

In the thematic development stage of analysis, KES and RLB used a mix of data charts and visualizations in Dedoose, including descriptor codes to compare across different categories (e.g., site, participant type), coupled with in-depth review of codes and coded excerpts, to create analytic memos and initial themes. Importantly, we used Dedoose’s normalization function to account for different amounts of data across the three sites which yields proportionately comparable metrics and data displays to examine cross-site or cross-role differences [23]. KES and RLB then triangulated findings across the varied data sources in Dedoose with site-specific contextual data to more fully describe and compare sites on palliative care structures, supports, and social norms related to palliative care in general and specialty palliative care specifically, and clinician-level attitudes, role identity, and orientation to care. These analyses resulted in a number of themes and contributed to the development of a middle range theory for palliative care integration (described below). Throughout the process, the analysis team shared triangulated findings for review and discussion with the entire research team. We also shared preliminary final results with the study advisory board which included site PIs (SE, LCH, GBR) and other experts in the field of specialty palliative care as a type of member checking and validation [24].

## Results

We conducted 62 clinician (lasting between 45 and 60 min) and 27 leader interviews (lasting between 30 and 45 min), 410 observations of inpatient and outpatient encounters, and 7 non-encounter-based site observations, such as tumor boards and care conferences (Table 1). Variation in observations across sites was due to the systematic reduction of observations at Site B to lessen the data collection burden for field staff while still ensuring thematic saturation. In addition, there was non-systematic reduction of observations at Site C due to the declaration of the COVID-19 pandemic and associated visitor restrictions which coincided with the first week of Site C’s onsite visit.

**Table 1:** Overview of Data Collection and Participants

Site	A	B	C	Total
Data Collected				
Number of Onsite Days	11	8	3	22
Leadership Interviews	7	11	9	27
Clinician Interviews	23	20	19	62
Observation Occurrences	41	22	10	73
Number of Observed Patients	228	134	48	410
**Clinician & Leader Characteristics**	**n = 26**	**n = 24**	**n = 18**	**n = 68**
Gender Female Male	12 (46%) 14 (54%)	12 (50%) 12 (50%)	7 (39%) 11 (61%)	31 (46%) 37 (54%)
Age (years) Average Range	44 33–59	47 32–64	41 34–63	44 32–64
Race Asian Black White Another Race	6 (2%) 0 (0%) 15 (58%) 3 (1%)	2 (8%) 2 (8%) 15 (63%) 2 (8%)	1 (6%) 1 (6%) 9 (50%) 5 (28%)	9 (13%) 3 (4%) 39 (57%) 10 (16%)
Hispanic/Latino	1 (3%)	1(4%)	0 (0%)	2 (3%)
**Observed Patient Characteristics**				
Gender Male Female	181 (44%) 216 (53%)	106 (47%) 119 (52%)	59 (44%) 64 (48%)	16 (33%) 32 (66%)
Age (years) Average Range	60 18-93	60 21-89	59 23-93	60 18-93
Race (observed) White Black Another Race	186 (45%) 150 (37%) 8 (2%)	85 (37%) 108 (47%) 6 (3%)	71 (53%) 28 (21%) 4 (3%)	30 (63%) 14 (29%) 0
Hispanic “Yes”	10 (2%)	4 (2%)	4 (3%)	2 (4%)
Surprise Q “No” *	47%	35%	34%	40%

* Observed clinicians were asked if they would be surprised if patient died within the next 12 months

Table 2 provides an overview of site characteristics and context related to specialty palliative care staffing capacity, processes, and structures, some of which have been theorized to be important for its integration [5]. While sites had similar numbers of patients with cancer, Sites B and C had more specialty palliative care personnel and interdisciplinary specialty palliative care teams. All sites had outpatient palliative care clinics, and Sites B and C also had inpatient palliative care units, but Site C’s team was only partially co-located with outpatient oncology. Site A had no automatic referral criteria or triggers for specialty palliative care while Sites B and C had a few. Lastly, all three sites tracked EOL metrics, but Site B tracked and shared more EOL metrics (e.g., with institutional leaders) compared to Sites A and C.

**Table 2:** Site Context Related to EOL and Palliative Care Delivery

Site	A	B	C
Location	Urban, Mid-West	Urban, Southeast	Urban, Southeast
New oncology patients per year	~ 5000	~ 5350	~ 4750
Size of specialty palliative care team (FTE) at time of study	3-outpatient 1-inpatient	4-outpatient 9-inpatient	5-outpatient 8-inpatient
Palliative care team is interdisciplinary*	No	Yes	Yes
Outpatient palliative care clinic*	Yes	Yes	Yes
Inpatient palliative care unit*	No	Yes	Yes
Palliative care co-located with outpatient oncology*	Yes	Yes	Partial
Palliative care automatic referral criteria or trigger (e.g., clinical care pathways for involvement)*	None	Some	Some
Number of EOL metrics tracked by organization (e.g., chemo in last two weeks of life)	Low	High	Medium
Level of sharing/ use of EOL metrics beyond administration	Low	Medium	Low

EOL = end of life; FTE = full time equivalent

*Indicator of palliative care integration in Hui 2015 [5]

**Fig. 2:**
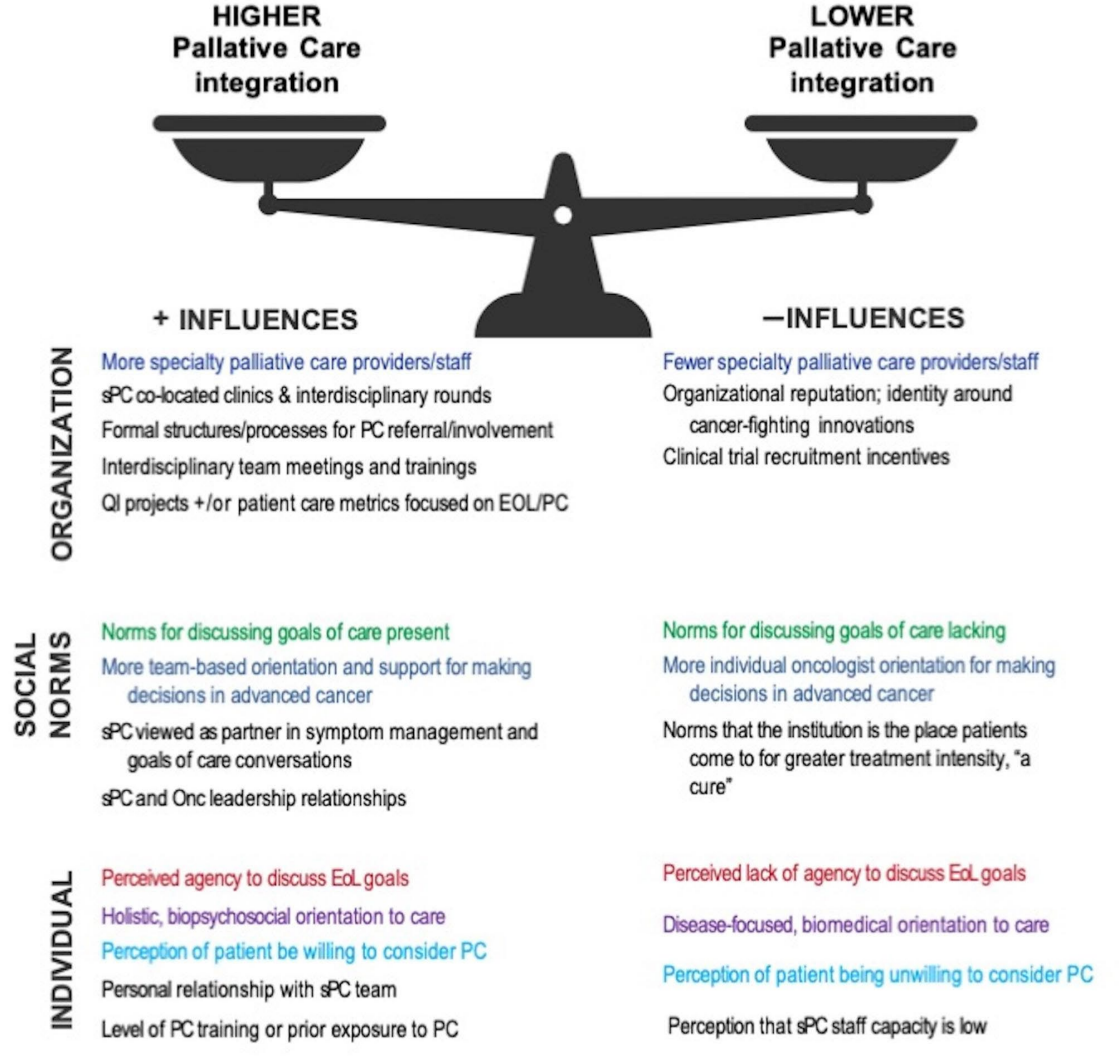
Main Themes: Influences on specialty palliative care (sPC) integration in advanced cancer care. NOTE: same color indicates one theme that can have an either positive or negative influence

Overall, when considering results of our qualitative analyses, we found that integration of specialty palliative care services in advanced cancer care was associated with a complex interaction of positive and negative influences within organizational, social norm, and individual clinician level factors. Figure 2 presents our main themes related to positive and negative influences for specialty palliative care integration in advanced cancer care, with the scale illustrating that more or less of these different influences can lead to higher or lower integration of palliative care which we describe more next.

### Organization-level influences

Seven main influences (see Fig. 2) at the organizational level emerged as supporting or discouraging specialty palliative care integration. In terms of positive influences, more versus fewer specialty palliative care professionals (FTE) and co-located clinics and personnel presence (e.g., clinical rounding) in both the outpatient and inpatient settings increased knowledge and utilization of specialty palliative care services.

*SITE C [specialty palliative care Director]: The opening of the palliative care unit that was the branch point that took palliative care services here to the next level, and we never looked back, because we owned real estate [on the outpatient side].*

*SITE B [GI Medical Oncologist]: Well, we are lucky in GI oncology that [palliative care clinician] is with us on Fridays and we do a lot of concurrent visits with him and his palliative care team, which I think has been a huge help since he’s been with us… and we have plenty of anecdotal experience of the people in the ER [emergency room] who we’ve gotten straight from the ER to inpatient hospice because of that collaboration and that allows us to have him involved in their care earlier.*

Staffing and presence is necessary, but not sufficient to ensure specialty palliative care integration. As noted by an oncologist at Site C, clinician schedules need to be coordinated to ensure overlap with specialty palliative care.

*Site C (Med/Hematology Oncologist). It’s really hard to get together in the same room and talk to the patient, or even for us to like get together and talk about this. Generally we kind of pick up the phone, brief conversation, or through notes in the chart……because, they’re all pulled into so many directions, and we’re all like different people going in different times. The campus is so big, and we’re kind of stretched all over. So it’s kind of hard to get together with all these problems.*

Formal structures or processes for referral or engagement of specialty palliative care were also supportive to engage specialty palliative care. Examples included standardized depression and distress screens at Site C which triggered specialty palliative care referrals, and formal diagnosis-based policies for initiating its referral at Site B (e.g., Stage IV lung cancer).

*Site C [Hematology Oncologist]: We have rolled out, through our cancer service line, our palliative counseling program, for our HEME/ONC patients, specifically on our HEME/ONC unit, a trigger to do distress screening on admission. And so if they score high enough on their distress screening, they will automatically trigger our palliative counseling service.*

Another organization-level influence was related to the presence and use by sites of joint meetings and trainings with specialty palliative care leaders and oncologist groups, including grand rounds. All three sites shared examples or were observed doing these, but site A had fewer mentions and was more limited in capacity due to fewer specialty palliative care personnel.

Sites varied in the degree to which EOL metrics were tracked or related QI initiatives. Site A tracked basic EOL metrics (e.g., chemotherapy in the last two weeks of life, hospice admission in last three days of life) and participated in related national and regional quality consortia. However, interviews revealed that tracking was focused more on revenue (e.g., value-based payments). In contrast, Sites B and C tracked more EOL metrics, including those related to specialty palliative care utilization (e.g., specialty palliative care patient volume). However, no site disseminated results beyond leadership and specific quality-oriented operational staff (e.g., to frontline clinical teams or other stakeholders).

Negative influences at the organizational level included perceptions of fewer specialty palliative care clinicians/staff and their capacity, a strong identity around cancer treatment innovations, and more disease-directed clinical trial recruitment, which appeared to compete with or delay specialty palliative care engagement, particularly at Site A. The following quotes from a radiation oncologist at Site A and an intensivist pulmonary medicine physician from Site C illustrate the orientation and social pressure related to clinical trial enrollment.

*SITE A: [Radiation Oncologist] It’s my job to make sure they understand what they need to understand … it’s just about seeing where that patient is mentally and emotionally, and letting them know that we are available to offer standard of care [options] or treatment on a clinical trial. We’re a designated cancer center and so we put a lot of patients on clinical trials that explore improved ways of doing things.*

Site C: [ICU/Pulmonary Medicine] *It’s happened several times where the residents are actually criticized about wanting to do advanced care planning and getting their [patient’s] goals or wishes stopped because I think that that comes against the, not only the inpatient oncologist, but the patient’s primary oncologist. So they’re feeling like they’re navigating two strong attendings who both want this person to be pushed into this clinical trial, and talking about advanced care planning is going to make them at risk to not enroll in the study.*

### Social Norm-level influences

Social norms across the organization and within clinical teams or departments played an important role in influencing EOL care delivery and engagement of specialty palliative care services. As noted in the introduction, social norms are informal (often unspoken) rules, attitudes and beliefs upheld in practice among a majority of group members. Recognizing that norms are shaped and interact with organizational and individual-level factors, we identified five main influences to specialty palliative care integration related to social norms, including those around: (a) goals of care conversations; (b) decision making for advanced cancer care and whether team- or collaborative decisions were typical versus decision-making by individual providers; (c) norms around how specialty palliative care as a practice was viewed in relation to oncology care; (d) norms associated with the social familiarity and networking between oncology and specialty palliative care providers; and (e) perceptions of what patients with advanced cancer did or didn’t want for their care by coming to that site.

Sites varied in their norms around goals of care conversations, and whether these conversations were embedded as part of routine care or viewed as an action of last resort. While goals of care conversations could promote either more or less ongoing treatment, they offered opportunities to discuss patient’s or their caregiver’s questions and wishes. How and when these conversations happened, to what extent providers opened up the goals conversation beyond immediate treatment decisions, and the degree to which specialty palliative care was engaged to support or lead these conversations differed based on site norms and by individual provider beliefs, which will be discussed in the next section. Implications of delaying or not discussing goals of care may lead to situations described by a hospitalist at Site A:

*SITE A [Internal Medicine Hospitalist] What we have noticed is [that for] most of the patients, the concept of hospice is introduced very late at a very advanced stage of cancer, and to the point that many of the patients say, “Well, I’ve met my oncologist a week ago and I was told everything was fine.“ So it’s difficult to bring up hospice at that point because if someone has been told everything is fine a week or 10 days ago and now you’re telling that the patient has less than three months or two months left, that’s where the problem comes from.*

A second way in which social norms varied at sites relates to the participants involved with decision-making. In some cases, care teams collaborated, whereas in others the primary oncologist decided whether to have goals of care conversations or involve specialty palliative care. While all sites collaborated somewhat in their decision-making, there was more deference at Site A to the primary oncologist. This resulted in more hospitalists, intensivists, and other inpatient consultants caring for patients whose conditions were rapidly deteriorating feeling they could not make the decision to initiate EOL discussions without first deferring to the patient’s primary oncologist.

SITE A [Hospitalist responding to patient vignette in interview]: *It looks like she has stage four cancer. So, I think a frank conversation about end of life should happen, but the protocol here is like they [primary oncologists] don’t want us to do it.*

Examples of a culture of team-based collaboration were most prominent at Site B, followed by Site C. This included evidence of higher involvement of non-physician staff, including nursing, social workers, or case managers, advocating for EOL-type conversations or referrals to specialty palliative care, and more support between clinicians advising each other about care decisions.

*Site B: Observation of 60-yr old white male inpatient with metastatic melanoma in the ICU with the intensivist and oncology attendings. The wife and son are in the room, but the presentation by an intern is very biomedically oriented and does not include the wife or son. The nurse navigator kind of chips in, and she’s sort of speaking for the wife. She said, “Mrs. So-and-So is asking those really scary, bigger picture questions. Would it be helpful to involve palliative care in this patient’s care?“ Both the attendings say, “Yes.“ The oncologist seems to be a little bit wary, but says, “Yeah. The doctors can imagine many different possible outcomes of his cancer care here. He could get better and leave the hospital and get treatment, or he could not get better. So, it’s helpful for palliative care to have you think about those different potential scenarios and what he might want in those situations.”*

*SITE B [specialty palliative care clinician]: And so if I’m worried that a patient is in a different place and that they are to have a change in their clinical status, or if I do my initial assessment and they sort of say they have some gaps in knowledge where they feel like they haven’t totally been informed about what’s going on with their cancer and what their prognosis might be, my personal practice is to then send a message, or do a text message, or an Epic message to the oncologist and say, “Hey, this is my first time meeting the patient. I didn’t feel comfortable or like I had enough information to really delve into some of the prognostication, but they were really interested in doing that. Can we schedule a time sometime in the next few weeks where we could come back and do that together so that I can hear what you’re saying to them and help facilitate a discussion about what that looks like and what’s most important to them?“*

A third way in which social norms influenced specialty palliative care involvement was in the way specialty palliative care was viewed in relation to and in the context of oncology care. At some sites, specialty palliative care was viewed more as a partner beyond strictly providing EOL care, such as assisting with more comprehensive symptom management or intervening at times of challenging family dynamics affecting care decisions. This in turn broadened the scope for when specialty palliative care was considered and engaged in care.

*Site B: [RN oncology administrator] “But it (palliative care) goes, from a pain management, a supportive service perspective and then obviously, end of life. Kind of that transitional thing. We really try to encourage our physicians to think about this at the beginning of treatment in terms of, what is the patient going to need? And that it should be really embedded within that constant thought… with advanced care planning and so forth.”*

Similarly, we identified normative influences associated with the degree to which specialty palliative care leaders had built relationships across the organization, especially with oncology leaders and teams. Where social familiarity and networking among specialty palliative care clinicians, leaders and oncology teams was greater, there was a tendency for earlier engagement of specialty palliative care in supporting patients with advanced care and greater overall awareness of it and appreciation for the ways that it could contribute to care delivery.

Lastly, we found that social norms based around perceptions of what patients with advanced cancer did and didn’t want with their care influenced specialty palliative care involvement. When the predominant view was that patients were seeking greater treatment intensity and “cures” when coming to the site for care, we saw more mentions of seeking fourth-line treatments, clinical trial enrollment, and last ditch efforts (often resulting in hospitalization at end of life) and delayed or absent specialty palliative care involvement. Clinicians at Site A talked the most about the patient population at their site specifically choosing them for the chance of more specialized treatment options and potentially “being cured.”

### Individual clinician-level influences

Six primary themes related to specialty palliative care integration at the individual clinician level were: (1) perceived agency to discuss EOL goals, (2) orientation to their clinical role in delivering care, (3) perception of a patient’s willingness for referral to specialty palliative care and/or hospice, (4) level of training and previous exposure to specialty palliative care, (5) perception of specialty palliative care staffing capacity at the site, and (6) personal relationship with specialty palliative care team member. These influences were observed alone and in varying combinations with positive or negative influences to specialty palliative care integration. For example, the degree to which clinicians expressed a sense of agency to initiate goals of care conversations, particularly when a patient wasn’t “their” patient, emerged as an important influence on specialty palliative care integration. The negative effect of this factor was most prominent for clinicians working in inpatient settings at Site A, where individual clinicians expressed frustration in not being able to initiate conversations because they were not the primary oncologist.

Clinician orientation to their clinical role and identity also influenced specialty palliative care integration. Clinician orientation was observed to be either predominantly a disease-focused biomedical orientation (negative influence) or a more wholistic biopsychosocial orientation (positive influence). For instance, during interactions with patients and caregivers, clinicians with more of a biomedical orientation focused on a narrower set of goals of care specific to symptom management and disease-directed treatment options such as later line chemotherapy options or clinical trial enrollment and did not introduce other non-treatment options or assess quality of life goals for their patient. A number of clinicians, including the following two medical oncologists, described this biomedical orientation.

Site C [Hematology Oncologist] *I think there’s a little bit of sort of what diseases people treat and how comfortable… How much they do in their own clinics, as well. There are just also some people who sort of philosophically are more aggressive in terms of really wanting to push, push, push, push, push through.*

SITE A [Radiation Oncologist]: *So I think a lot of physicians, in general, over treat patients and don’t feel comfortable having what essentially end up being end of life discussions.*

When clinicians viewed goals of care more holistically and with greater acknowledgement of patients having limited life expectancy, patient-clinician conversations were more likely to include discussion of a wider range of options including specialty palliative care, hospice, and community-based resources.

Similarly, perceptions of patient willingness to consider palliative care, including hospice, also influenced whether or not clinicians began to talk about palliative care related topics or made specialty palliative care referrals. In this study, perceptions of patient willingness were often influenced by experiences with or assumptions and stereotypes of patients based on their racial background, religion, ‘culture’, and/or education and SES.

*SITE A [Medical Oncologist]: So their demographic background. Yeah. That does influence things. I mean, it doesn’t influence things as far as affect decisions. But certain situations, I think, certain ethnicities are much more accepting of hospice. I find that Caucasians are much, much more accepting of hospice and feel better when end of life situations than other ethnicities. It’s more difficult in African-Americans and Arab-Americans. We don’t have a lot of Hispanics here, so I haven’t experienced it in that population.… I think at least in the Arabic community, they don’t believe in, they feel like hospice is giving up, and that’s not okay. So it’s hard to change their mind or kind of the idea or what they think of hospice.*

*Site C [Medical Oncologist]: The Southern black community also invokes religion a lot, but there’s a little bit of an inherent distrust of the, “You say I’m going to die in two or three months and that this isn’t curable. Yeah. I don’t really believe you.“ I mean it doesn’t mean that they don’t come and see us and we don’t still do their treatment, but there’s still this underlying distrust that’s there. And I think that just makes these conversations.... If they don’t trust what you’re saying, it inherently makes it much more difficult.*

Other individual-level positive influences included having a personal relationship with someone on the specialty palliative care team and amount of training about palliative care or prior exposure to it.

*Site B (Pulmonary/Critical care Intensivist): A lot of our young faculty have had dedicated palliative care experiences through their training. They are now on faculty and training others.*

Lastly, perceptions of lack of specialty palliative care team capacity or availability negatively influenced specialty palliative care engagement and referrals.

### Middle Range Theory for specialty palliative care integration

As noted previously, each of our three sites had different combinations of contextual factors related to specialty palliative care (Table 2) and positive and negative influences on specialty palliative care integration shown in Fig. 2. Nevertheless, when triangulating our findings, we identified an overall middle-range theory [25–27] for specialty palliative care integration. First, formal organizational structures and specialty palliative care capacity were important for creating a base infrastructure to support and facilitate specialty palliative care integration. For instance, given fewer specialty palliative care clinicians at Site A (context), availability to conduct interdisciplinary rounds (organization level) is less and influences perceptions of specialty palliative care availability (individual clinician). More importantly however, was the combination of formal organizational structures with positive social norms, such as valuing the importance of earlier goals of care conversations and a broader perception of the role of specialty palliative care as a care team partner *before* the last few days of life. **Our principal theory is that the presence of strong and favorable organizational structures and supports in combination with supportive social norms reduces the influence of individual clinician orientation and actions (or inactions) on specialty palliative care integration in advanced cancer care.** We do not suggest that individual clinician influence is not important for specialty palliative care integration or that they can’t have a positive influence; rather that clinician-level attitudes and orientation has less influence on specialty palliative care integration when strong organizational and social norms for it exist.

**Fig. 3:**
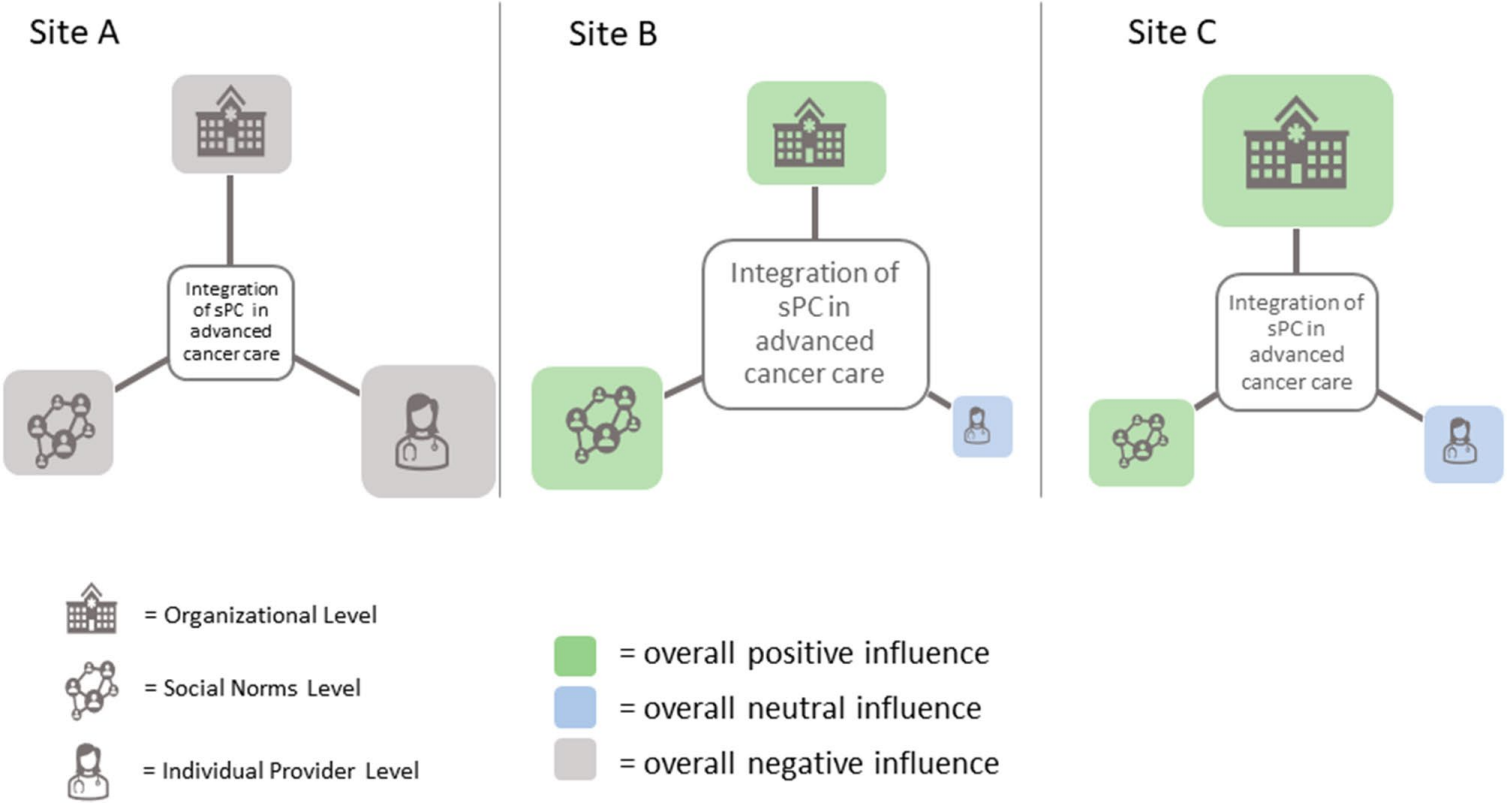
Interactions of specialty palliative care influences by site (size of boxes indicate the amount of influence within each level; color indicates direction of influence per legend below)

Figure 3 illustrates our theory through an overall comparison of specialty palliative care integration at the three sites based on the interaction of contextual factors and the three levels of influences. The size of the boxes representing the levels of influence (e.g., organizational) indicate the number of factors from that level influencing specialty palliative care integration while the color indicates the overall direction of the influence (green-more positive for specialty palliative care integration; blue neutral, gray more negative). As seen in Fig. 3, Sites B and C had higher amounts of specialty palliative care integration (center box) overall compared to Site A. Participants from Site C reported the most organizational level specialty palliative care and EOL resources including staffing capacity and formal structures for specialty palliative care involvement. Site B had no formal screening process or policy but did have other organizational supports including more multidisciplinary clinics and rounds, joint meetings, QI projects and several specialty palliative care-specific referral triggers. Site B also had developed stronger, more favorable social norms to specialty palliative care integration compared to Sites A and C, which in combination with the organizational structures had the overall effect of increasing specialty palliative care referrals and engagement at that site. When presented with the advanced cancer patient vignettes during interviews, Site B clinicians were the most likely to talk about the decisions and discussions related to advanced care planning and shifting from disease-directed treatments to managing symptoms and engaging specialty palliative care clinicians. Site A had far fewer formal structures for EOL planning and specialty palliative care engagement at the organizational level and very limited specialty palliative care capacity (only two specialty palliative care physicians at the time of our site visit). In addition, there was high organizational identity for developing and testing cancer treatment innovations combined with social norms for oncologist primacy in decision making. Many narratives indicated a lack of agency by non-oncology clinicians to initiate goals of care conversations. In combination, these factors created more pressures for higher EOL treatment intensity at Site A and much greater reliance on individual clinician actions for initiating specialty palliative care services, if they were utilized.

### Limitations

It is important to note that we may not have observed or had sufficient data on important interactions and potential influences on specialty palliative care integration given that we were only able to include three sites. In addition, our middle range theory, while informed by extensive data and triangulation of data sources across our three sites, may or may not be generalizable to other sites and we encourage other researchers to test the theory. However, we posit that the more positive influences at each level (organizational structures and resources, social norms, and individual clinicians), the more likely a site will be to have earlier and better integration of specialty palliative care in the care of patients with advanced cancer.

## Discussion

Our study supports a number of indicators of palliative care integration summarized by Hui [5], such as multi-disciplinary palliative care teams, symptom screening and referral criteria, outpatient and inpatient palliative care clinics, and co-location with oncology, suggesting that these are important strategies to implement for better integration. However, ​the indicators are mostly structural and do not acknowledge influences related to social norms and individual clinicians’ orientation to palliative care which our study highlights. We found integration varied at our three sites depending on their mix of influences across all three levels. However, our middle range theory suggests that a strong organizational base for specialty palliative care, which includes personnel, systems, physical space, and rules or policies supporting or promoting palliative care, combined with social norms is necessary to support more integration. We don’t know the order or direction of the influence (e.g., if norms supporting palliative care contribute to getting financial support and resources for specialty palliative care that builds the organizational base or vice versa), but we posit that the presence of both produces a reinforcing cycle that leads to better integration of specialty palliative care and encourage others to test this theory.

Unfortunately, for the foreseeable future, building a strong organizational base for specialty palliative care will be challenging due to lack of certified, specialty palliative care clinicians [28, 29]. Even our sites with seemingly adequate-sized specialty palliative care teams (B and C) indicated that they were not able to meet all eligible patients’ needs. Proposals to address this palliative care capacity gap include creating training for midcareer “palliative care champions” who “would bridge the gap between” certified, specialty palliative care and other clinicians to deliver palliative care [30], more research on “optimal organization and allocation of limited resources in specialty palliative care to close the gap between the workforce and patient need” [31], and efforts to define and require training on “primary palliative care (skills that all clinicians should have)” [32].

We believe that all these additional strategies are essential to close the palliative care capacity gap but caution that equal attention should be paid to creating favorable social norms for palliative care. That is, since individual non- palliative care clinicians need to play a key role in delivering primary palliative care or referring to specialty palliative care ​both favorable social norms and organizational structures are necessary. Thus, we encourage sites interested in promoting palliative care integration to assess strategies and communication which acknowledge social norm influences and potentially address them, such as assessing who is “allowed” to initiate goals of care conversations or feedback from patients on whether their goals were ascertained, when and by whom.

## Conclusion

In their 2017 article, Kaufmann and Kamal propose that integration of palliative care and oncology care requires “reimagining how optimal integration occurs” by aligning “with the financial and practical needs of a busy oncology practice that must view integration as adding value and not as an additional task” [33]. Given current evidence for specialty palliative care, including patient and caregiver outcomes, multi-level influences highlighted in this study, and continued gaps, we argue that integration is only achieved through overall attention to organizational supports and social norms which value palliative care.

## Electronic supplementary material

Below is the link to the electronic supplementary material.

Supplementary Material 1

Supplementary Material 2

Supplementary Material 3

## Data Availability

The data that support the findings of this study are available on request from the corresponding author Karen E. Schifferdecker. The data are not publicly available due to them containing information that could compromise research participant privacy and consent.

## List of abbreviations

EOL: End-of-life
ICU: Intensive care unit
NCI: National Cancer Institute
NQF: National Quality Forum
