# Supplementary material for: Structure and integration of specialty palliative care in three NCI-designated cancer centers: a mixed methods case study
Source: BMC Palliat Care. 2023 May 16;22:59. doi: 10.1186/s12904-023-01182-9 (PMC10185464; doi:10.1186/s12904-023-01182-9)
Supplement: Supplementary file 1 — Supplementary Material 1 [file 12904_2023_1182_MOESM1_ESM.docx]

**ADDITIONAL FILE 1**

**SEMI-STRUCTURED LEADERSHIP INTERVIEW GUIDE**

**INTRODUCTION**

*Participants will have already consented to be interviewed*

Hi, I’m [INTERVIEWER NAME]. Thank you for taking the time to talk with me today.

The purpose of the site visit that we are doing is to observe how care for patients with advanced cancer is provided at your site. The purpose of this interview is to get some background on [SITE or “in your service line”] and how you do things so we can better understand your processes.

We prefer to audio record so we can focus on listening rather than note taking. If you agree to be audio-recorded, the recording will be transcribed without any names or other identifying information. Do I have your permission to audio record our conversation?

*Procedure > BEGIN RECORDER*

**DEMOGRAPHIC INFORMATION**

1. Could you tell me how you self-identify your race?
2. And are you of Hispanic ethnicity?
3. Could you start by telling me a bit about your work at [site]? how long you’ve been working at [SITE] and your primary role?

**STRUCTURES FOR ADVANCED CANCER CARE – *Select questions according to interviewee role***

**🡪  *Clinical Leads, e.g., Cancer Center Director***

I would like to switch to a few general questions around some of the structures related to advanced cancer care here at [SITE].

1. How is the inpatient service organized at your site for caring for patients with lung, breast or other solid tumor cancers when they’re admitted?
   1. How is the outpatient service at your site organized?
2. How is the care of advanced cancer patients coordinated here at [site], for example, meetings or other kinds of processes for reviewing and discussing advanced cancer cases among providers?

**🡪** ***Palliative Care Director***

I would like to switch to a few general questions around how palliative care is organized here at [SITE].

1. How many palliative care providers are at [SITE]?
2. What other kinds of staff or providers are considered part of [SITE’S] palliative care team?
   1. When you think about everyone who is involved in palliative care here, would you say you can fully meet the demand for palliative care services here, partially meet the demands, or are barely able to meet the demands?
3. When and how does the palliative care team typically get involved with a patient here at [SITE]?
   1. What is the typical time between receiving a palliative care referral and when a patient is first seen?
   2. Would you describe the process for involving palliative care as being a formal process that everyone knows or is it more informal?
   3. Who typically initiates the referral process?
   4. Are there specific triggers for a palliative care referral?
   5. How well would you say the referral process works here?
4. How do providers learn about how to engage with your team here at [SITE]?
5. How would you describe the culture around palliative care here? What is your sense of how other providers think about or value palliative care?
6. How much is the palliative care team integrated into cancer care here at [site]?

**🡪** ***Chief Nursing Officer, Social Work or Case Manager***

I would like to switch to a few general questions around the processes and resources for patients nearing end of life here at [SITE].

1. When and how is the decision made to start the conversations with patients and family around end of life care options, including palliative care and/or hospice?
   1. Who typically has these conversations with the patient and family?
2. What is the process for referring and enrolling a patient in hospice?
   1. Is this the same for both inpatients and outpatients? If not, how does it differ?
3. Could you give me a sense of the availability of hospice-related services for your patient population such as residential or home-based services?
   1. Would you say there are enough options available or not enough?
4. Are there patients who have more trouble accessing hospice services?
   1. (If yes) Can you tell me more about those patients and the reasons they have more trouble?

**GENERAL INFORMATION**

1. When you think about care for patients with advanced cancer at your site or in your service line, how would you say you are doing?

*Depending on how they answer (more positive or more negative), explore more what they are doing well OR what they think they are not doing well.*

1. What are things you are doing particularly well OR Can you tell me more about what you think you need to improve?

*Probe about things relevant to the informant’s role (if not mentioned):*

- *advance care planning*
- *palliative care referral*
- *hospice referral*
- *chemo decision making*
- *acute care: ED visits/hospitalizations/ICU admissions for patients near the end of their life*
- *navigation/case management*

1. How do you know how you are doing related to caring for patients with advanced cancer? What kinds of things does your site track or share related to this?

*Probe about things like % of patients who have an ACP, palliative care referral, hospice referral, chemo use, ED visits, hospitalizations, and ICU admissions at end of life*

1. How would you describe the way that advance care planning, including completing Advance Directives, is done at [site]?

How much does [site] focus on having a copy of a patient’s living will / AD on file?

1. Does [SITE] have any written protocols or guidelines for any aspect of advanced cancer care, like Advance Directives or end of life planning?

How much would you say these are being followed or used on a day-to-day basis?

*If not much:*

What would you say are the primary reasons these are not being used?

*If some or a lot:*

What would you say are the primary reasons that these are being used?

**QUALITY MEASURES**

1. So I know there are many different quality measures out there now and I would like to ask you about some end-of-life quality measures endorsed by ASCO and the National Quality Forum. This is not a test—I’m just curious if you have heard of these measures that include things such as receiving chemo in the last two weeks of life and late referrals to hospice?
2. How well do you think your site is doing related to the NQF end-of-life quality measures?
3. When is performance data shared? (cadence, who/how, blinded/unblinded)
4. Thinking about your (site or service line), how many of your providers and staff would know how your site is doing related to these measures?

**DISPARITIES**

1. What kind of conversation is there at (site, service line) about health disparities in advanced cancer care? (probe re: racial/ethnic disparities in particular, guidelines, programs/interventions to address, performance data by race)

**CLOSING**

1. What else haven’t I asked about regarding your site’s care for patients with advanced cancer that you think I should know about?

*Reiterate Confidentiality and Express Thanks*

If there is nothing else that you can think of, I want to thank you very much for taking the time to speak with me. If you have any questions or think of other things you would like to let me know about, please contact us. This has been enormously useful, so thank you once again for your time and your cooperation, it is deeply appreciated.
