# Supplementary material for: Structure and integration of specialty palliative care in three NCI-designated cancer centers: a mixed methods case study
Source: BMC Palliat Care. 2023 May 16;22:59. doi: 10.1186/s12904-023-01182-9 (PMC10185464; doi:10.1186/s12904-023-01182-9)
Supplement: Supplementary file 2 — Supplementary Material 2 [file 12904_2023_1182_MOESM2_ESM.docx]

Additional File 2

semi-structured PROVIDER Interview GUIDE

**1. INTRODUCTION**

*Participants will have already signed study consent form to be shadowed/interviewed*

Hi, I’m [INTERVIEWER NAME]. Thank you for taking the time to talk with me today.

The purpose of this interview is to better understand your approach to patient care, specifically for patients with advanced cancer and how you might work to achieve the best outcome. Your answers will be confidential and discussed only among our research team. Although we may touch on some uncomfortable topics, please understand that this is a judgement-free interview, and I would appreciate your honest thoughts. If there are any questions that you prefer not to answer, or you would like to stop the interview at any point, please let me know.

If you agree to be audio-recorded, the recording will be transcribed without any names or other identifying information. Do you mind if I audio record our conversation?

*Procedure > BEGIN RECORDER*

**2. BACKGROUND INFORMATION**

I would like to collect some information on your background to start.

Could you tell me about your role at [SITE]?

How long have you worked here?

*[ask below questions also if you are not sure if we have demographic information for the provider]*

What is your race? *(list categories)* Amer Indian/Alaskan, Asian, Black, Hispanic, Hawaiian/Pacific, White

Are you Hispanic or Latino?

How old are you now?

What year did you graduate from medical school?

Which type of residency did you complete? What year did you complete it?

Did you complete fellowship training? If so, what type of fellowship? What year did you complete it?

# 3. Vignettes

Thank you. Now I would like to ask you several questions to understand your experiences and thoughts, starting with a few vignettes—I’ll start with Vignette number [XX].

# *Present interviewee with two cases, individually. Give interviewee time to review each case prior to questioning.*

# What are your initial impressions of this case? How would you manage this patient?

# *Optional Prompts:*

- - - What do you think your next steps would be and how would you anticipate discussing your next steps with the patient?
    - Thinking about your next steps in this case, how are they similar to or different from what your colleagues might do?
    - What would happen if you didn’t do [X] and how would that affect the patient?
    - What would your colleagues think, do or say if you didn’t do [X]?
    - Are there particular things about this patient that made you think you should [X]?
    - How might this patient’s expectations affect your approach?

# *[When you give provider second vignette say]* Thank you. Now I would like to have you look at Vignette number [XX].

# 4. MENTAL MODELS

# I really appreciate you going through these vignettes. Thinking in general…

- - - What are the kinds of things you think about when deciding how to manage a patient with advanced cancer?
    - Can you describe a recent (or memorable) case which involved caring for an advanced cancer patient where you experienced anxiety or discomfort about a decision, but you were ultimately satisfied with how things went?
      - What led you to end up feeling satisfied?
    - Can you describe a recent (or memorable) case which involved caring for an advanced cancer patient where you experienced anxiety or discomfort about a decision, and you were ultimately not satisfied with how things went?
      - What led you to end up feeling unsatisfied?

# 5. SITE and PATIENT FACTORS (race, ethnicity, socioeconomic, cultural)

I want to explore a few more things in terms of what might influence advanced cancer care specifically here at [SITE]. Some of my questions may explore areas that feel sensitive or uncomfortable but I hope that you will be as honest as you can be so I can fully understand your perspectives. Please remember, everything you share is completely confidential.

- - - First, based on your experiences and observations and thinking about patients with advanced cancer, how do patients’ backgrounds influence the way providers here think or communicate about their treatment or care plans?
      - (If not mentioned as part of patients’ backgrounds) How would you describe the care for patients from different races or ethnicities who come here for advanced cancer care in terms of treatment or care plans?
        - In your opinion what things contribute to this (their description of the care) being the case here?
    - If you think about your own feelings or thoughts when working with patients from different races or ethnicity than yourself, what are the things that come to mind? How is that for you?

Can you recall a time (in working with patients of different race/ethnicity than you) when you recognized a bias that you have?

If yes, can you tell me the experience and what you learned from it?

**6. CLOSING**

What else is relevant to your thought processes in caring for advanced cancer patients that we haven’t discussed?

If there is nothing else that you can think of, I want to thank you very much for taking the time to speak with me and to share your experience. And I want to reiterate once again that all of your comments will be treated as strictly confidential. The information you have shared will be combined with the responses from other interviews for analysis and will not be associated with you personally in any way. This has been enormously useful, so thank you once again for your time and your cooperation, it is deeply appreciated.

***TIPS FOR INTERVIEWING***

*General Prompts:*

- *“Anything else?”*
- *“Can you explain/elaborate?”*
- *“Can you tell me more?”*
- *“Can you tell me what you mean by…?”*
- *Rephrase their response*

# *General Topics:*

# *ACP*

# *Chemotherapy use*

# *Acute care use*

# *Palliative care*

# *Hospice*

# *ICU admission, life-sustaining treatments, code status*

# *Rationalizations (implicit and explicit motives)*
