# Supplementary material for: Structure and integration of specialty palliative care in three NCI-designated cancer centers: a mixed methods case study
Source: BMC Palliat Care. 2023 May 16;22:59. doi: 10.1186/s12904-023-01182-9 (PMC10185464; doi:10.1186/s12904-023-01182-9)
Supplement: Supplementary file 3 — Supplementary Material 3 [file 12904_2023_1182_MOESM3_ESM.docx]

**Additional File 3**

**Patient Vignettes 1 and 2 used in clinician interviews**

**Vignette 1**

**JH (gastric cancer)**

**CC:** Shortness of breath and anxiety

**HPI:** JH is a 71-year-old man with metastatic gastric cancer who was hospitalized early this morning from a skilled nursing facility (SNF) with shortness of breath. At the time of his transfer he was recovering from a previous hospitalization for sepsis, during which he was admitted to the ICU for mechanical ventilation and dialysis. Prior to that hospitalization he had been receiving second-line palliative chemotherapy, with uncertain effectiveness. He has been at the SNF for the last week, where he has been unable to gain weight or increase his physical activity. Last night he developed a fever and shortness of breath, and he was transferred to the emergency department.

In the ED, blood cultures were drawn, intravenous levofloxacin and azithromycin were administered, he was given a nebulizer, and he was started on oxygen (6L by nasal cannula). He was admitted to the inpatient floor a few hours ago, and his wife is in the room with him now. JH felt symptomatically improved initially after the nebulizer treatment, but his difficulty breathing has worsened over the last hour and he seems increasingly anxious.

**PMH:** Hypertension, coronary artery disease, diabetes, chronic renal insufficiency

**PSH:** No prior abdominal or thoracic surgeries.

**SH:** Nonsmoker, occasional EtOH, no illicit drug use. Retired high school teacher. He is Catholic. He is married and lives with his wife (Emma). He has a daughter who lives in Hawaii.

**Home medications:** Lisinopril, aspirin, atorvastatin, insulin

**Physical Exam:**

Temp: Temperature 38.3; BP 90/55 mm Hg; RR 30/min; HR 125; O2 90-92% on 50% face mask

General: Moderate respiratory distress with face mask

**Labs:**

ABG: PH 7.35; PCO_2_ 46; PO_2_ 62.

**Imaging:**

- Chest x-ray showed metastatic cancer and possible pneumonia.
- Recent CT (from one week ago) showed progressive metastases in the lungs and liver.

**Vignette 2**

**Identification:** VF is a 68 year-old woman with a recent diagnosis of pancreas cancer.

**Cancer history:** VF was previously in excellent health, but she developed diabetes about four months ago. Six weeks ago she began to experience low back and abdominal pain, and she has lost 20 lbs in the past month. Recently she developed jaundice, which prompted a CT scan of the abdomen and pelvis. This showed a large pancreatic head mass obstructing the common bile duct. No distant metastases were seen, and the radiologist described the pancreatic tumor as “borderline resectable”. An ERCP was performed, with biopsy of the pancreas and stenting of the common bile duct. The biopsy showed adenocarcinoma, and jaundice has resolved with stenting.

**PMH/PSH**: Osteoarthritis.

**Meds**: ibuprofen, as needed

**Social history**: She is widowed and lives alone. She has two sons who live far away. She is a non-smoker and does not drink alcohol.

**Exam:**

Vital signs: BP 110/74. HR 105. Pain 6 of 10 (abdomen and low back).

General: Cachectic, but in no acute distress.

Abdomen: No palpable abdominal masses. Diffuse abdominal tenderness without rebound or guarding.

**Performance status:** ECOG 3 (Capable of only limited self-care, confined to bed or chair 50% or more of waking hours)

**Pertinent lab findings:**

Albumin 2.6 (L); total bilirubin 1.4 (H); CA 19-9 689 (H).

🡪 She was recently admitted to the inpatient medicine service for pain, nausea and vomiting, and failure to thrive. With titration of pain medications and antiemetics, her pain and nausea are better controlled now, however she is unable eat or ambulate without assistance. She has now been in the hospital for 5 days without improvement in strength or caloric intake. You have been consulted for further management recommendations.

She is tearful and tells you she has never been so overwhelmed, weak and exhausted. However, she says “I’m a fighter, not a quitter” and “with Jesus, anything is possible.” She then asks “what comes next?”
